# COVID-19 Infection as a Possible Cause of Ogilvie’s Syndrome

**DOI:** 10.7759/cureus.32345

**Published:** 2022-12-09

**Authors:** Sarmad Pirzada, Zarak H Khan, Amanda Mahoney, Ali A Mankani

**Affiliations:** 1 Internal Medicine, Trinity Health Livonia Hospital, Livonia, USA; 2 Gastroenterology, East Carolina University, Greenville, USA; 3 Internal Medicine, Dow University of Health Sciences, Karachi, PAK

**Keywords:** sars-cov-2 infection, gastro intestinal, viral infection, covid 19, ogilvie's syndrome

## Abstract

Ogilvie’s syndrome is defined as acute dilatation of the colon in the absence of mechanical obstruction. Even though the precise mechanism is unknown, studies have suggested its association with autonomic nervous system dysfunction. Some of the common causes include infections, orthopedic surgery, renal failure, electrolyte disturbance, and narcotic use. Viral causes are considered to be rare; however, it is a well-known fact that viral infections can cause autonomic dysfunction. A few cases have been reported discussing the incidence of Ogilvie’s syndrome in the setting of severe acute respiratory syndrome-coronavirus-2 (SARS-CoV-2). We present a unique case of Ogilvie’s syndrome in a patient who initially presented with respiratory manifestations and subsequently developed acute colonic pseudo-obstruction.

## Introduction

Ogilvie's syndrome is defined as distension of the colon without any evidence of mechanical obstruction [1]. Many studies describe the association of Ogilvie's syndrome with viruses such as cytomegalovirus (CMV) and varicella-zoster virus (VZV) [2,3]. However, only a few cases have been reported to discuss its relationship with severe acute respiratory syndrome-coronavirus-2 (SARS-CoV-2). SARS-CoV-2 is an airborne viral disease primarily manifesting in the respiratory system. However, it has also been shown to infect the gastrointestinal tract through its viral receptor angiotensin-converting enzyme 2 (ACE-2) expressed on enterocytes of the ileum and colon, resulting in symptoms such as diarrhea, nausea, vomiting, and abdominal pain [4]. The involvement of these receptors has also been recently associated with acute pancreatic and liver injury in patients with SARS-CoV-2 infection [5,6]. Here, we discuss a unique case involving a patient who initially presented with respiratory manifestations of SARS-CoV-2 disease requiring prolonged mechanical ventilatory support and subsequently developed Ogilvie's syndrome during hospitalization. Our case was initially presented at the World Congress of Gastroenterology, organized by the American College of Gastroenterology, in October 2020 [7].

## Case presentation

**Figure 1:**
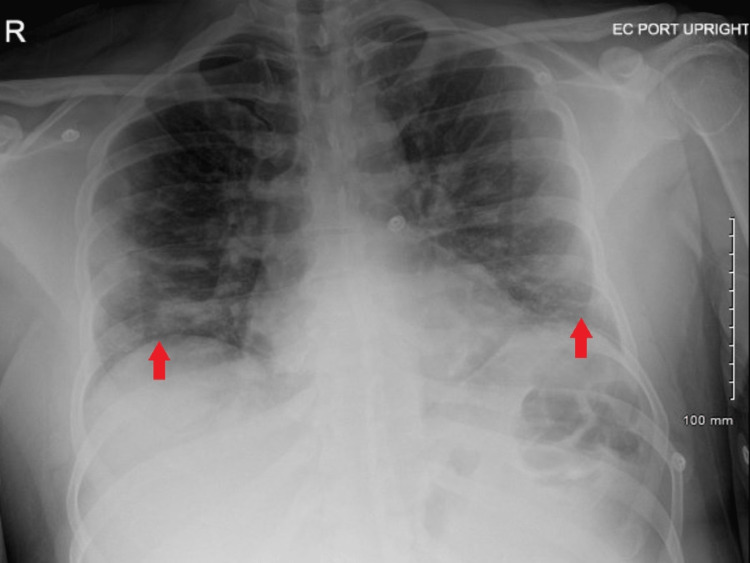
Chest X-ray Findings consistent with vague ill-defined opacities at the lung bases bilaterally, raising concern for atypical bilateral pneumonia (red arrows).

A 38-year-old male with a history of hypertension, bipolar disorder, and tobacco use was presented to the emergency department with eight days of flu-like symptoms, including myalgias, fevers, dyspnea, cough with intermittent clear phlegm, and two days of blood-tinged sputum. He tested positive for SARS-CoV-2, and his chest X-ray was consistent with atypical pneumonia (Figure 1).

**Figure 2:**
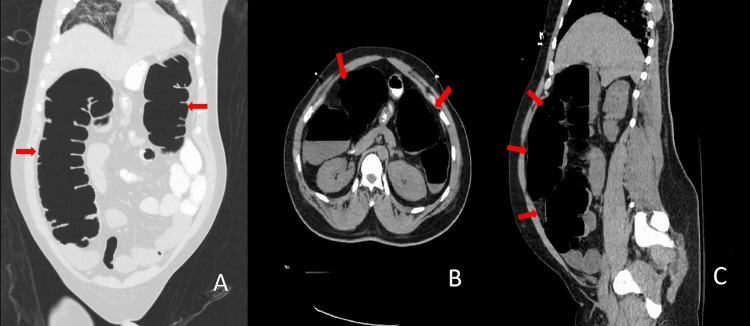
Abdominal CT scan: (A) coronal view; (B) axial view; (C) sagittal view Different views of abdominal CT scan demonstrate dilated loops of large bowel (red arrows).

Later, during the hospitalization, he developed generalized weakness due to decreased oral intake. He was initially admitted to the medical floor with SARS-CoV-2 pneumonia, lymphopenia, and mild elevations of aspartate aminotransferase (AST) and alanine aminotransferase (ALT). During his course on the general medical floor, he was treated with five days of azithromycin and hydroxychloroquine and started on methylprednisolone and zinc supplementation. He developed tachypnea and hypoxia refractory to oxygen supplementation on hospital day (HOD) 6. Therefore, he was intubated and transferred to the ICU. While in the ICU, the patient developed acute kidney injury requiring intermittent continuous renal replacement therapy. Enteral feeding was initiated on HOD 7. On HOD 12, he was noted to have increasing abdominal distension. Therefore, enteral feedings were held. An X-ray of his abdomen was consistent with gaseous distension of the colon. Administration of lactulose, methylnaltrexone, and enemas resulted in a slight liquid bowel movement. An orogastric tube was placed to suction and decompress his stomach to prevent aspiration. Given his lack of improvement, a CT scan of the abdomen was done, which showed distended small and large bowel loops without evidence of obstruction (Figure 2A-2C).

Ogilvie's syndrome was suspected. Therefore, he was given two doses of neostigmine, resulting in a bowel movement. He did not require decompressive colonoscopy. The patient started having regular bowel movements with enemas and continued using neostigmine. His ventilator requirements gradually decreased. He was extubated to a heated high-flow nasal cannula on HOD 23 and eventually transitioned to an oral diet. A repeat X-ray of his abdomen showed improvement in colonic distension. He maintained normal oxygen saturations and was medically stable for discharge on HOD 35.

## Discussion

Although SARS-CoV-2 is more commonly associated with respiratory symptoms, gastrointestinal manifestations of the illness have been increasingly reported. A multicenter cohort study performed across nine hospitals in Massachusetts assessed the prevalence of gastrointestinal symptoms in patients with laboratory-confirmed SARS-CoV-2 infection. The results showed that 61.3% of the patients with SARS-CoV-2 reported at least one gastrointestinal symptom at presentation, the most common of which included loss of appetite, diarrhea, and nausea [8].

While the pathogenesis of SARS-CoV-2-associated gastrointestinal complications is incompletely understood, the mechanisms thought to increase the risk of gastrointestinal tract involvement involve the ACE-2 receptors. The ACE-2 receptor is the host receptor for the SARS-CoV-2 virus. While these receptors are abundantly present in type 2 alveolar cells of the lungs, they are also abundantly found in the alimentary canal, predominantly the small and large intestines [9]. In some cases, the gastrointestinal symptoms of SARS-CoV-2 may even precede respiratory manifestations of SARS-CoV-2 infection, suggesting that the small intestine may also be an important site of cellular entry for the virus [10].

Our case report is of a patient with active SARS-CoV-2 infection requiring prolonged ventilatory support, which developed Ogilvie's syndrome later during hospitalization. To date, only a few other cases have been reported that draw a connection between Ogilvie's syndrome and SARS-CoV-2 infection. One example is the case involving a 60-year-old female with a recent SARS-CoV-2 disease, while ours involved a 38-year-old male with an active infection [1]. Another study reported two elderly patients developing acute colonic pseudo-obstruction, while not being on any ventilatory support, in the setting of an active SAR-CoV-2 infection [11]. This suggests that there can be the development of Ogilvie's syndrome in patients with an active or a recent SARS-CoV-2 infection, with or without being on ventilatory support. However, as seen in our case, regardless of whether the disease is current or active, the best pharmacological line of treatment is neostigmine therapy which showed results shortly after initiation.

For SARS-CoV-2 patients with increased abdominal distension and prolonged constipation, the best course of action is to perform physical examinations and obtain abdomen radiographs every 12-24 hours to rule out the possibility of Ogilvie's syndrome [1]. A water-soluble enema should be performed before radiography to rule out a mechanical obstruction in instances where distension and gas do not occur throughout the entire colon [12]. In patients with massive abdominal distension and a cecal diameter greater than 9 cm, it is essential to start treatment promptly to avoid the complication of a perforated cecum [13].

The management of Ogilvie's syndrome includes conservative measures (electrolyte replacement, bowel rest, nasogastric suction) and interventions like acetylcholinesterase inhibitor therapy such as neostigmine or colonic decompression [13]. Some cases may even require surgery [13]. However, most patients respond to conservative management, which should be the first line of treatment before any intervention. Supportive therapy can also involve cessation of oral intake or enteral feeding, as done in our case. Patients who do not respond to conservative management should undergo pharmacological intervention with neostigmine. A meta-analysis by Valle et al. has proven that one dose of neostigmine can be 89.2% effective in resolving acute colonic pseudo-obstruction [14]. Colonic decompression or surgery should only be used as the last line of treatment.

## Conclusions

There is still much that we do not know about the novel coronavirus. While several studies have been conducted to understand the pathogenicity of SARS-CoV-2, many consequences of the infection are still unknown. Our case report shows that patients with SARS-CoV-2 may also develop non-respiratory symptoms such as Ogilvie's syndrome during their disease. Our case also highlights the importance of considering and evaluating Ogilvie's syndrome in SARS-CoV-2 patients experiencing prolonged constipation and increased abdominal distension to facilitate timely diagnosis and management. Further studies are required to shed more light on the pathogenesis and incidence of Ogilvie's syndrome in patients infected with SARS-CoV-2.
